# Human rhinoviruses prevailed among children in the setting of wearing face masks in Shanghai, 2020

**DOI:** 10.1186/s12879-022-07225-5

**Published:** 2022-03-14

**Authors:** Ran Jia, Lijuan Lu, Shu Li, Pengcheng Liu, Menghua Xu, Lingfeng Cao, Liyun Su, Jin Xu

**Affiliations:** 1 Department of Clinical Laboratory, Children’s Hospital of Fudan University, Shanghai, 201102 China; 2 School of Laboratory Medicine and Life Sciences, Wenzhou Medical University, Wenzhou, 325035 Zhejiang China

**Keywords:** Human rhinovirus, Respiratory tract infection, Children, Molecular epidemiology, Wheezing

## Abstract

**Background:**

Human rhinovirus (HRV) is the predominant etiological agent of the common cold in children and adults. A recent study showed that the inhibitory effect of face masks on viral shedding of HRV was less prominent than that on other respiratory viruses. Considering that most Chinese people have worn face masks in public area since the outbreak of coronavirus disease 2019, we aimed to find out whether HRV prevailed among children in 2020 and demonstrate the details of the epidemiological features of HRV under such a special circumstance.

**Methods:**

We summarized the incidences of various respiratory virus infections in patients who visited the Children’s Hospital of Fudan University during 2018–2020, and genotyped HRV positive nasopharyngeal specimens collected from 316 inpatients and 72 outpatients that visited the hospital in 2020.

**Results:**

There was a major prevalence of HRV among children in the latter half of 2020, with a clear seasonality that HRV-As prevailed in summer while HRV-Cs in autumn. HRV-As were more prone to cause severe lower respiratory tract infections (LRTI), while HRV-Cs were closely associated with childhood wheezing. The predominant genotypes were A11, A28, A47, A82, A101, C40 and C43. Notably, A21, A82 and A101 took up larger proportions in severe cases than in non-severe cases.

**Conclusions:**

Our findings described a major prevalence of HRVs among children in 2020, which highlight the unique transmitting pattern of HRV and help to narrow the targets for antiviral strategies.

## Background

Human rhinoviruses (HRVs) are the leading cause of upper respiratory tract infections (URTIs) since its first isolation in the 1950s [1]. HRVs also cause pneumonia hospitalization in vulnerable people such as children, the elderly and those with underlying diseases. HRV-associated diseases pose great socio-economic burdens to the country annually [2]. However, given that HRV-infected people are usually manifest self-limited and mild symptoms or even asymptomatic, HRVs have long been afforded little attention and no antivirals or vaccines have been approved for HRVs up to now [3].

HRVs belong to the *Picornaviridae* family, and are single-stranded, positive-sense RNA viruses, indicating that it contains the sense strand of RNA as their genome which can be readily translated into proteins [4]. The genome is approximately 7,200 base pair (bp), including a single open reading frame (ORF) (~ 6500 bp), a 5’ untranslated region (UTR) (~ 650 bp) and a 3′ UTR (~ 50 bp) [5]. About 100 serotypes which were culturable in vitro were classified into HRV-As and HRV-Bs based on the similarity of partial genetic sequences in the 1990s. Afterwards, at the beginning of the 2000s, researchers identified at least 50 more new HRV strains which couldn’t be cultured and were classified into a unique species now named as HRV-C [2]. So far, more than 160 HRV genotypes have been identified [6].

After the outbreak of coronavirus disease 2019 (COVID-19), most people have developed the habit of wearing face masks in public area in order to inhibit the transmission of respiratory pathogens. However, Leung et al. quantified the amount of respiratory viruses in exhaled breath of participants with acute respiratory illnesses and found that wearing medical face masks significantly reduced the RNA level of influenza viruses and coronaviruses (OC43 and NL63) in respiratory droplets or aerosols, but not in HRVs [7], suggesting that the inhibiting effect of face masks may be less effective in HRV transmission. Hence, we conducted this research to further figure out whether HRVs could still spread among children in spite of the popularization of face masks and meanwhile demonstrate the details of the epidemiological features of HRVs. The findings in this study will expand the knowledge of HRV epidemiology and arouse people’s attention to HRV’s unique transmission pattern under such a special background.

## Methods

### Patients and sample collection

A total of 316 nasopharyngeal aspirates from inpatients with lower respiratory tract infection (LRTI) hospitalized in the Children’s Hospital of Fudan University in Shanghai from June 2020 to November 2020 were collected in this study. All the inpatients were diagnosed with LRTI supported by symptoms and radiographic changes and were defined as HRV positive after routine screening for common respiratory viruses including respiratory syncytial virus (RSV), adenovirus (AdV), influenza A and B viruses (IAV and IBV), parainfluenza virus type 1 (PIV-1), PIV-2, PIV-3, human rhinoviruses (HRV) and human metapneumovirus (MPV). For HRV screening, RNA from respiratory samples were extracted using a magnetic beads-based nucleic acid extraction system NP968-C (Tianlong Technology, China) according to the manufacturer’s instruction. Then a one-step real time quantitative polymerase chain reaction (RT-qPCR) kit (Land medical, China) with primers targeting the *5′UTR* (263 bp) of HRVs was used to detect HRV RNA. The remaining viruses and mycoplasma were detected using an immunofluorescence assay kit (Diagnostic Hybrids, USA). Briefly, nasopharyngeal aspirates were centrifuged and the cell pellet was fixed in acetone. A mixture of fluorescein-labeled monoclonal antibodies directed against the target viruses were added onto the cells, followed by an incubation of 30 min at 37 °C. A Mounting Fluid containing glycerol was added onto the stained cells and then a coverslip was placed on the prepared cells. The cells were examined using a fluorescence microscope (Nikon, Japan). Isolation and culture of bacterial and fungal pathogens were carried out according to the routine microbiology examination and diagnosis. Bacterial and fungal strains were identified using VIETEK automated bacterial analyzer (France) or MALDI-TOF/MS mass spectrometry (Bruck, France).

A total of 703 nasopharyngeal swabs from outpatients with URTI who visited the hospital during June 2020 to November 2020 were collected randomly and screened for HRV by RT-qPCR. The randomization was done as follows: first, one staff member covered all the information of the patients on the swabs with a blank tag paper. Then another staff member was asked to choose the swabs randomly to avoid biases in patients’ gender, age, and illness.

LRTIs are illnesses that affect the respiratory system below the throat. The severity-based classification of the patients was performed by experienced clinicians according to the World Health Organization (WHO)’s latest definition of severe LRTI cases [8, 9]. Briefly, a child of any age with danger signs (e.g. cyanosis, seizures, lethargic/unconscious, unable to drink/breastfeed, respiratory failure) were defined as severe LRTI cases [8–10]. All experiments in the study were carried out in accordance with relevant guidelines and regulations. The study was reviewed and approved by the Ethics Committee of the Children’s Hospital of Fudan University on Feb 2020 (Approval Number: 202027).

### HRV genotyping

For genotyping, the extracted RNA were reverse transcribed and amplified using a nested RT-PCR strategy. HRV molecular subtyping was performed using primers targeting the *VP4/VP2* regions (540 bp) of HRVs as reviewed in a previously published paper [11]. To increase both the sensitivity and efficiency of genotyping, we used a modified nested PCR method [12]. Briefly, the reverse transcription and the first amplification step were performed using a one-step RT-PCR kit (Rui’an Biotechnology, China) with outer primers: VP-OS (5′-CCGGCCCCTGAATGYGGCTAA-3′) and VP-OAS (5′-ACATRTTYTSNCCAAANAYDCCCAT-3′). The second amplification step was performed using a Premix Taq kit (Takara, Japan) with inner primers: VP-IS (5′-ACCRACTACTTTGGGTGTCCGTG-3′) and VP-IAS (5′-TCWGGHARYTTCCAMCACCANCC-3′) [11, 13]. The amplification products were sequenced by Sangon Biotech Co., Ltd., China, followed by subjection to phylogenetic analysis using MEGA software.

### Statistical analysis

Proportions for categorical variables were compared using the χ^2^ test or Fisher’s exact test. Independent group *t*-test was used for the comparison of means for continuous variables that were normally distributed. The Mann–Whitney U test was used for continuous variables not normally distributed. All statistical analyses were performed using GraphPad Prism software. Two-sided *p*-values of less than 0.05 were considered statistically significant.

## Results

### The prevalence of respiratory viruses

**Fig. 1:**
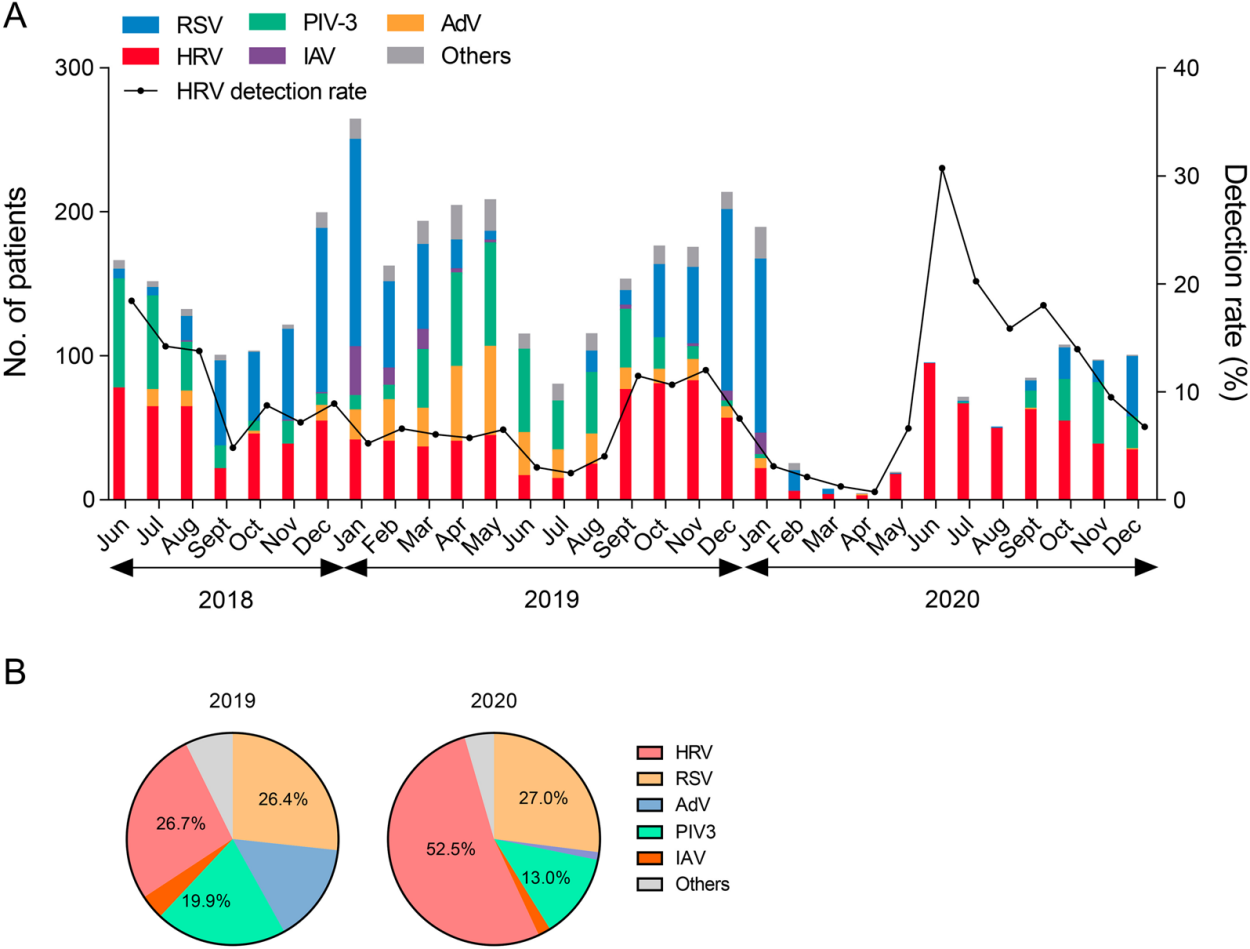
The prevalence of respiratory viruses in all the patients tested for the viruses in the hospital from June 2018 to December 2020. **A** Total numbers of the patients (including inpatients and outpatients) tested positive for the indicating respiratory viruses and the detection rate of HRVs in the specimens collected from the Children’s Hospital of Fudan University during June 2018 to December 2020 were displayed. The detection rate of HRV was the proportion of the HRV positive patients in the total patients whose nasopharyngeal samples were detected for the viruses in the indicated month. **B** Proportions of different respiratory virus in 2019 and 2020. *HRV* human rhinovirus, *RSV* respiratory syncytial virus, *AdV* adenovirus, *IAV* influenza A virus, *PIV-3* parainfluenza virus type 1. Others represented for the sum of PIV-1, PIV-2, IBV and human metapneumovirus (MPV)

**Fig. 2:**
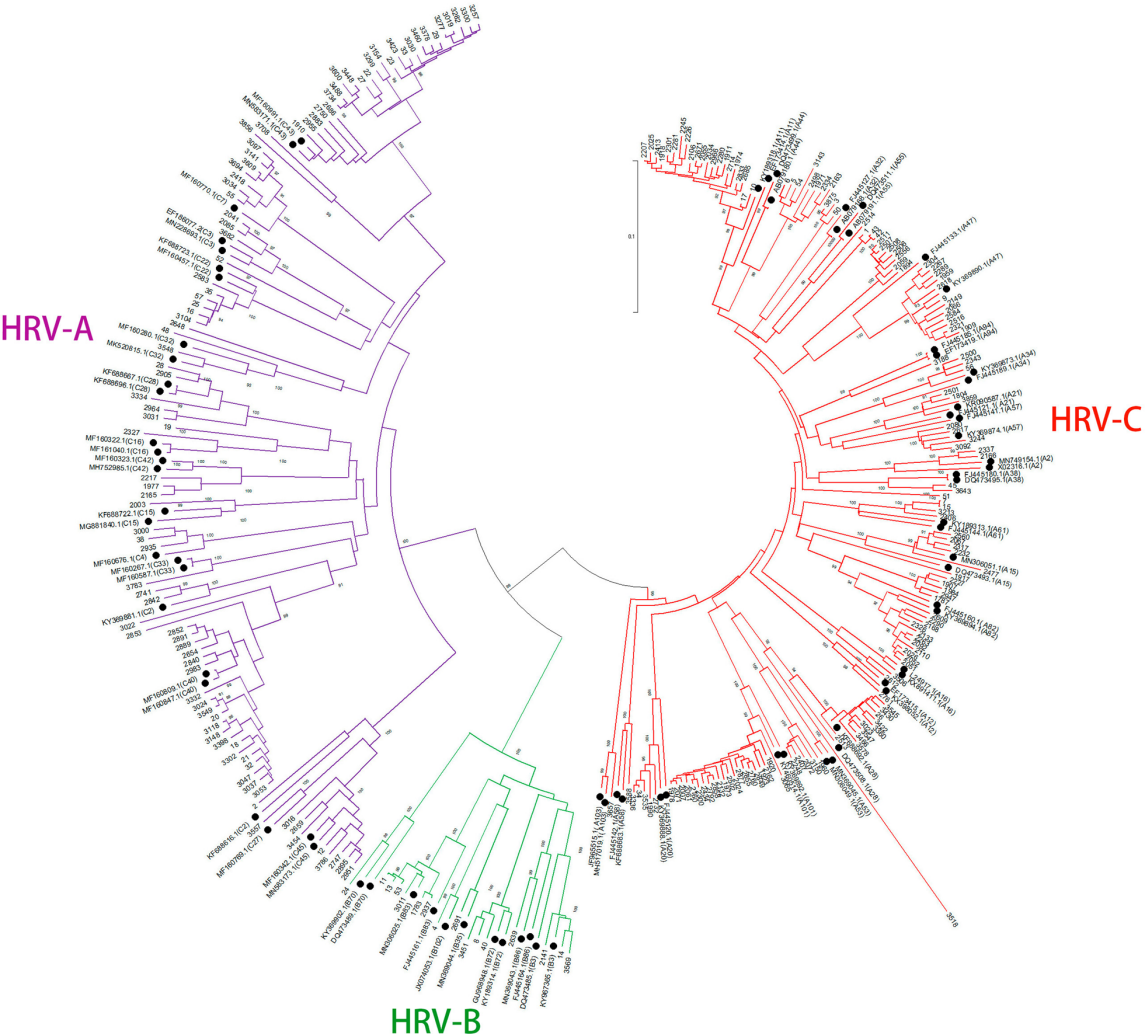
Phylogenetic trees of *VP4/VP2* gene sequences of HRVs. Phylogenetic trees were generated from manually trimmed 540 bp fragments using the neighbor-joining method and branch supported with 1000 bootstrap iterations using MEGA software. Bootstrap values were shown on tree nodes. Study sequences were identified by accession number. Reference sequences from GenBank were identified by accession number (HRV type) with black dots. Purple branches, HRV-As; Green branches, HRV-Bs; Red branches, HRV-Cs

A total of 4481 patients were tested positive for at least one of the following viruses as RSV, AdV, IAV, IBV, PIV-1, PIV-2, PIV-3, MPV and HRV in the Children’s Hospital of Fudan University from June 2018, when the hospital started the HRV test for patients, to December 2020. Most respiratory viruses were barely detected after the outbreak of COVID-19 (Fig. 1A), but HRVs showed a remarkable increase in the middle of 2020 in June 2020 among the 3 years. The detection rate of HRV (HRV positive patients/total patients tested for the virus) also reached the climax (30.7%, 94/306) in June 2020. There was a mild increase in HRV infection in September 2020. PIV-3s and RSVs also increased gradually but were much less prominent than HRVs. The proportion of HRVs in the total virus positive cases was 52.5% in 2020, which was much higher than that in 2019 (26.7%) (Fig. 2B). Collectively, these data indicated that there was a major prevalence of HRV in the year of 2020.

### Molecular epidemiology of HRVs

A total of 316 nasopharyngeal aspirates from HRV positive inpatients during the epidemics of HRVs in 2020 were collected and 82.3% (260/316) were successfully genotyped. We also randomly collected 703 nasopharyngeal swabs from outpatients with URTI during the same period of the inpatients, among which 10.2% (72/703) were determined as HRV positive and 90.3% (65/72) were successfully genotyped.

**Fig. 3:**
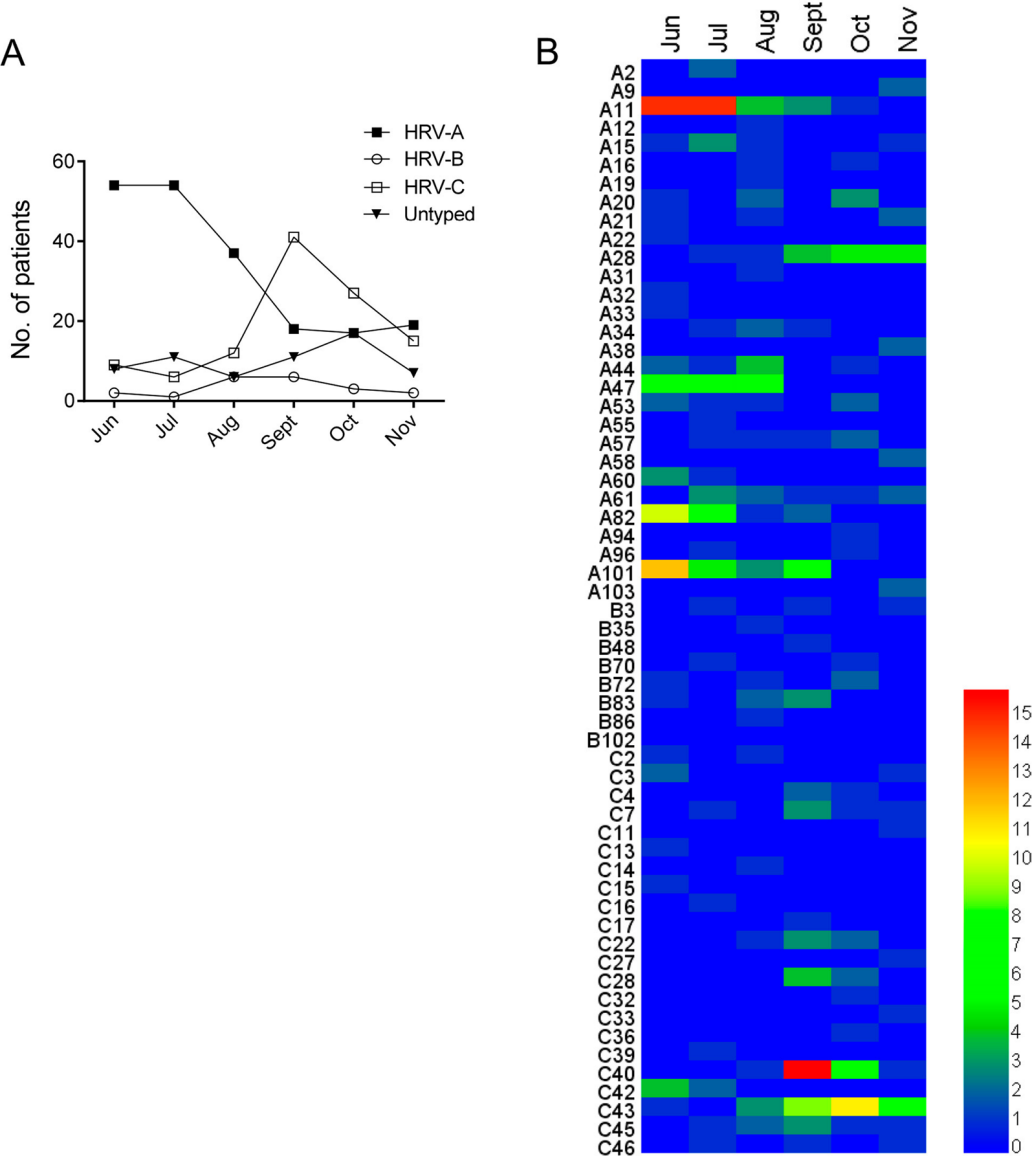
The seasonality of HRV genotypes. **A** The numbers of patients infected with different HRV species were shown by month from June 2020 to November 2020. **B** The numbers of patients detected positive for the indicating HRV genotypes were shown by month from June 2020 to November 2020

**Fig. 4:**
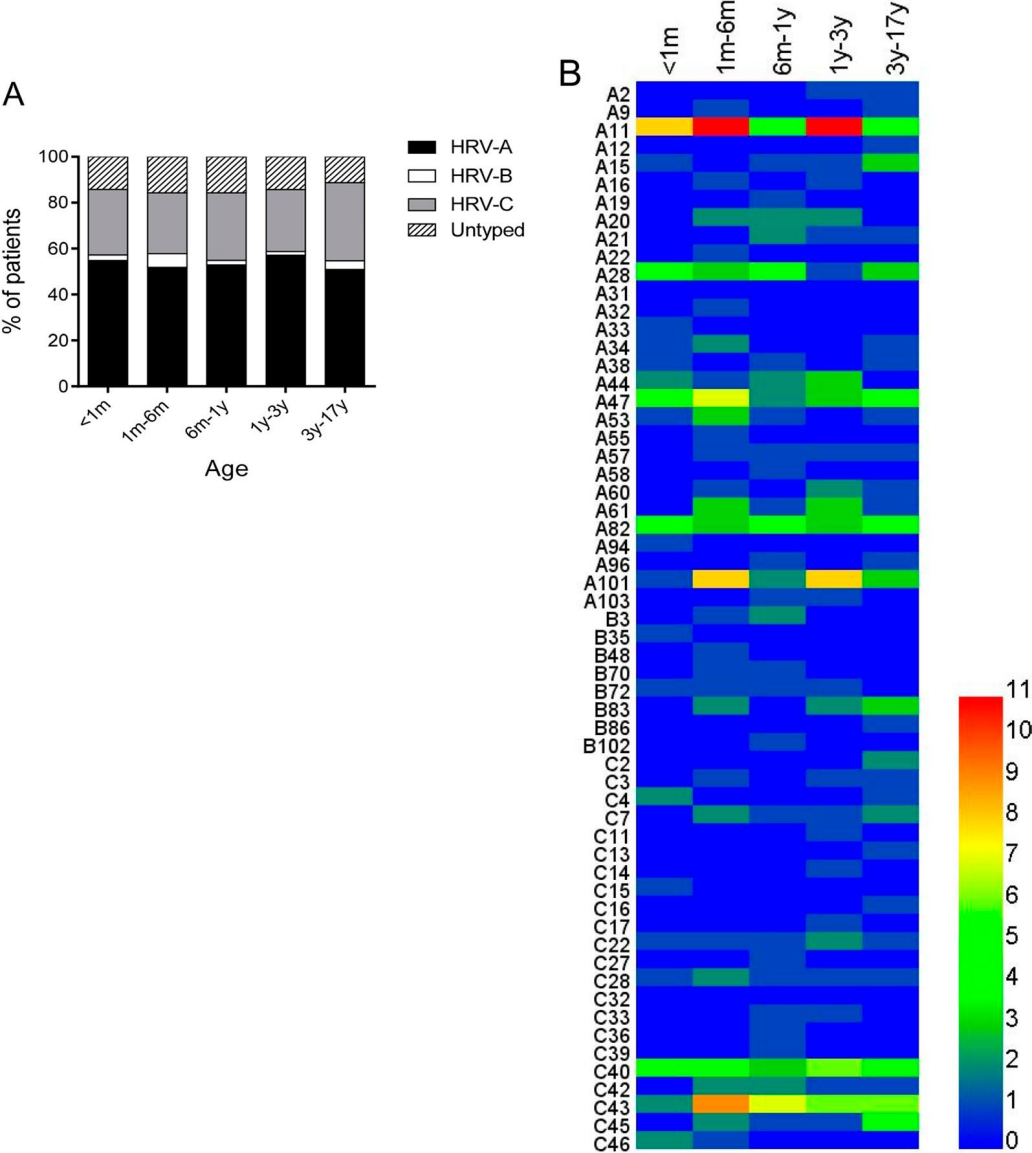
The distribution of HRV genotypes among patients of different ages. **A** The proportions of HRV species in different age groups from infants to adolescents. **B** The numbers of patients detected positive for the indicating genotypes were shown by age

The genetic variability of HRV genotypes in our data was very wide, as shown in the phylogenetic tree (Fig. 2). A total of 29 HRV-A genotypes, 8 HRV-B genotypes and 22 HRV-C genotypes were detected in the patients. HRV-A was the most frequently detected species both in the inpatients (51.6%, 163/316) and the outpatients (45.8%, 33/72), followed by HRV-C (27.2%, 86/316 for inpatients; 33.3%, 24/72 for outpatients) and HRV-B (3.5%, 11/316 for inpatients; 11.1%, 8/72 for outpatients). The monthly distribution of patients revealed that HRV-As mainly prevailed in the summer (June to August) with A11, A47, A82 and A101 being the most frequent, while HRV-Cs quickly caught up in the autumn and peaked in September represented by C40 and C43. Notably, unlike most HRV-As, A28 mainly prevailed in the autumn (September to November) rather than summer (Fig. 3). HRVs infected more males than females both in the inpatients and the outpatients, with the male/female ratio being 1.34:1 and 1.4:1 respectively. 55.7% of total HRV-positive patients were infants under 1 year of age, and no discernable differences was found in the proportions of HRV species among different age groups (Fig. 4A). Also, the predominant genotypes appeared to be similar among children of different ages (Fig. 4B).

### HRV genotypes and clinical features

To find out the association between HRV and clinical characteristics, we collected the clinical information of patients including symptoms, co-infections and underlying diseases. Among the three species, HRV-Bs seemed to infect more females than males, although the number of HRV-Bs were small (Table 1). Notably, HRV-Cs appeared to be the most frequently detected species in the 46 patients with wheezing (60.9%, 28/46). 11.7% (23/196) of the HRV-A positive patients were defined as severe LRTI, which was much higher than HRV-B (0%, 0/19) and HRV-C (4.5%, 5/110), suggesting that HRV-A is more prone to cause severe illness.

**Table 1:** The clinical features of patients among the three HRV species

	HRV-A (n = 196)	HRV-B (n = 19)	HRV-C (n = 110)	*p*-value
Male	134 (68.4%)	6 (31.6%)	82 (74.5%)	0.0010*
Age, median (min–max)	7m26d (1d-17y)	2m14d (26d-16y)	9m13d (13d-17y)	0.3627
Hospital stays, median (min–max), days	9.5 (1–120)	10 (1–63)	9 (2–106)	0.8602
Symptom				
Fever	56 (28.6%)	9 (47.4%)	31 (28.2%)	0.2136
Cough	78 (39.8%)	9 (47.4%)	55 (50.0%)	0.2130
Wheeze	15 (7.7%)	3 (15.8%)	28 (25.5%)	0.0001*
Tachypnea	13 (6.6%)	0	6 (5.5%)	0.4890
Cyanosis	1 (0.5%)	0	1 (0.9%)	0.8572
Respiratory failure	12 (6.1%)	0	5 (4.5%)	0.4802
Severe LRTI	23 (11.7%)	0	5 (4.5%)	0.0382*
Underlying disease	142 (72.4%)	12 (63.2%)	75 (68.2%)	0.5673
Cardiovascular system	40 (20.4%)	3 (15.8%)	11 (10%)	–
Hepatobiliary system	30 (15.3%)	2 (10.5%)	14 (12.7%)	–
Immune deficiency	7 (3.6%)	1 (5.3%)	3 (2.7%)	–
Hemopoietic system	3 (1.5%)	0	5 (4.5%)	–
Co-infections	28 (14.3%)	0	18 (16.3%)	0.1672
*Klebsiella pneumoniae*	4 (2%)	0	1 (0.9%)	–
*Haemophilus influenzae*	2 (1%)	0	1 (0.9%)	–
*Pseudomonas aeruginosa*	2 (1%)	0	1 (0.9%)	–
*Streptococcus viridans*	2 (1%)	0	4 (3.6%)	–
RSV	2 (1%)	0	3 (2.7%)	–
PIV	1(0.5%)	0	2(1.8%)	–
AdV	2(1%)	0	1(0.9%)	–
*Mycoplasma urinolytica*	3(1.5%)	0	0	–
*Mycoplasma pneumoniae*	2(1%)	0	3(2.7%)	–

*HRV* human rhinovirus, *LRTI* lower respiratory infection, *RSV* respiratory syncytial virus, *AdV* adenovirus

*P < 0.05 was considered of significant difference among the three groups

Afterwards we classified the patients into three groups according to their disease severity, including the outpatients with URTI, the inpatients with non-severe LRTI or severe LRTI (Table 2). No significant differences were found in the age distribution among the three groups. Females were more likely to develop HRV-associated severe LRTI, with 64.5% (20/31) severe cases being girls. In addition to longer hospitalizations, severe LRTI cases were more likely to have a cough than the other two groups. Notably, the percentages of underlying diseases increased in sequence from URTI (33.3%), non-severe LRTI (62.4%) to severe LRTI (87.1%).

**Table 2:** The clinical characteristics of patients with different disease severity

	URTI (n = 72)	Non-severe LRTI (n = 285)	Severe LRTI (n = 31)	*p*-value
Male	42 (58.3%)	170 (59.6%)	11 (35.5%)	0.0350*
Age, median (min–max)	1y9m (5d to 14y)	7m5d (1d to 17y)	1y6m (3m27d to 7y)	0.1427
Hospital stays, median (min–max), days	–	9 (1–104)	19 (4–120)	0.0007*
Symptom				
Fever	29 (40.3%)	61 (21.4%)	6 (19.4%)	0.0031*
Cough	12 (16.7%)	112 (39.3%)	18 (58.1%)	< 0.0001*
Wheeze	5 (6.9%)	34 (11.9%)	7 (22.6%)	0.0790
Tachypnea	0	9 (3.2%)	10 (32.3%)	< 0.0001*
Cyanosis^a^	0	0	7 (22.5%)	< 0.0001*
Unable to drink/breastfeed^a^	0	0	1 (3.2%)	0.0031*
Respiratory failure^a^	0	0	17 (54.8%)	< 0.0001*
Seizure^a^	0	0	5 (16.1%)	< 0.0001*
Lethargic/unconscious^a^	0	0	2 (6.5%)	< 0.0001*
Apnea^a^	0	0	1 (3.2%)	0.0031*
Underlying disease	24 (33.3%)	178 (62.4%)	27 (87.1%)	< 0.0001*
Cardiovascular system	2 (2.8%)	44 (15.4%)	8 (25.8%)	–
Hepatobiliary system	1 (1.4%)	42 (14.7%)	3 (9.7%)	–
Immune deficiency	3 (4.2%)	6 (2.1%)	2 (6.5%)	–
Hemopoietic system	4 (5.6%)	3 (1.1%)	1 (3.2%)	–
Co-infection	–	42 (14.7%)	4 (12.9%)	0.9946
Virus	–	12 (4.2%)	2 (6.5%)	–
Bacteria	–	19 (6.7%)	3 (9.7%)	–
Fungi	–	4 (1.4%)	2 (6.5%)	–
Mycoplasma	–	8 (2.8%)	0	–

*URTI* upper respiratory infection, *LRTI* lower respiratory infection

*P < 0.05 was considered of significant difference among the three groups

^a^These symptoms were included in the criteria of severe LRTI cases

**Fig. 5:**
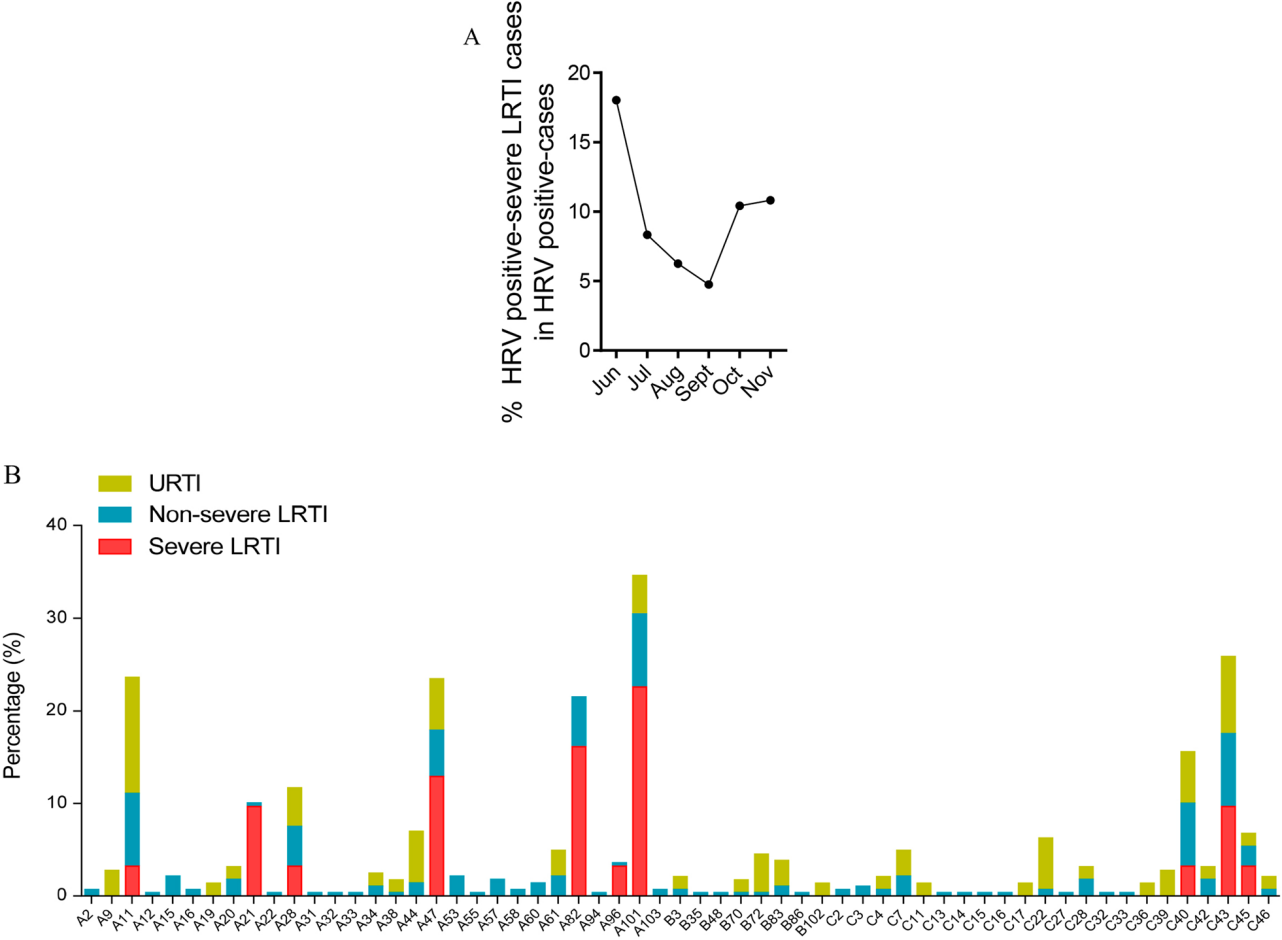
The diversity of HRV genotypes in patients of different severity. **A** The monthly distribution of severe LRTI cases in HRV-positive patients. **B** The percentages of different genotypes in patients of URTI, non-severe LRTI and severe LRTI groups respectively. *URTI* upper respiratory infection, *LRTI* lower respiratory infection

The percentage of severe cases in HRV positive cases showed an increase in October and November 2020 (Fig. 5A), despite that the detection rate of HRVs displayed a downward trend since September 2020 (Fig. 1A), indicating that the detection rate and severity of HRV infections did not correspond completely. The genotypes detected in severe group included A11, A21, A28, A47, A82, A101, C40, C43 and C45, all of which were also detected in non-severe LRTI patients (Fig. 5B). Notably, there were three genotypes which made up significantly larger proportions in severe LRTI cases than in non-severe LRTI cases, namely A21 (9.7% vs. 0.4%, *p* = 0.0004), A82 (16.1% vs. 5.3%, *p* = 0.0183) and A101 (22.6% vs. 7.7%, *p* = 0.0065). To be noted, A21 was the only genotype that was more frequently detected in severe LRTI cases (3 cases) than in non-severe LRTI cases (1 case), despite that the total number of non-severe LRTI cases was almost ten times of the severe LRTI cases.

**Fig. 6:**
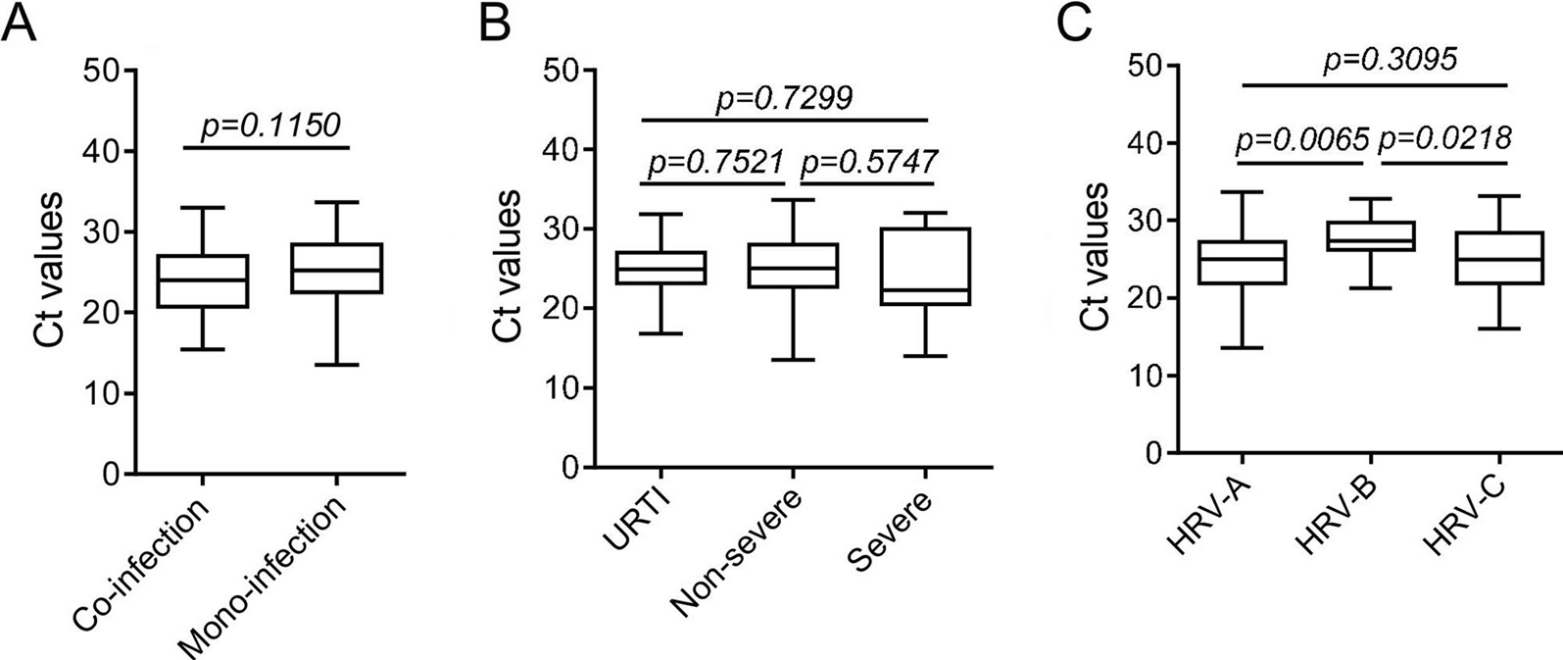
The Ct values of HRV positive samples. **A** The patients infected with HRV only or co-infected with other respiratory pathogens were divided into two groups, and their Ct values were acquired based on RT-qPCR. **B** The Ct values of HRV positive samples collected from patients classified based on their disease severity were shown. **C** The Ct values of HRV positive samples of different species were shown. *P* < 0.05 was considered of significant difference

To further investigate the viral load-associated factors, we collected the Ct values of HRV positive samples. In our data, the patients co-infected with other respiratory viruses showed comparable viral loads with those with HRV mono-infection (Fig. 6A). Also, the viral loads didn’t seem to be correlated with the disease severity (Fig. 6B). But HRV-Bs showed lower viral loads (higher Ct values) compared with the other two species (Fig. 6C), which was in line with its lowest prevalence and least possibility to cause severe illness.

## Discussion

HRV infections were mainly transmitted via aerosols generated by coughing, sneezing and nose [14], which is supposed to be effectively decreased by face masks. But the major HRV prevalence among children in 2020 indicates a weakened inhibitory effect of face masks [7]. Still, the unique transmitting pattern of HRV which enabled itself to escape from face masks deserves further investigation.

The majority of people wear disposable medical masks in public during the COVID-19 pandemic as WHO recommended, given that medical masks could help block large-particle droplets, splashes, sprays, or splatter that may contain viruses or bacteria [15]. But as we previously mentioned, the filtering effect of medical masks was insufficient to block HRV shedding [7], and the increased HRV infection during the COVID-19 pandemic has been reported in various countries [16, 17]. N95 masks, which are class II medical devices, are designed to achieve a very close facial fit and very efficient filtration of airborne particles. Unlike medical masks, N95 masks could confer much better protection and have been proved to effectively block viruses like the influenza virus and HRV [18, 19]. Hence, it is advisable for HRV positive patients to wear N95 masks in order to reduce transmission.

Generally speaking, non-enveloped viruses (eg, HRV and AdV) are more heat-resistant and could survive longer in a dry and acidic environment than enveloped respiratory viruses (eg, RSV, IAV, PIV, and CoV), which largely increases their chances of spread [20]. Also, it is very easy for children to touch contaminated surfaces/objects (fomites). Children usually couldn’t wash their hands timely and couldn’t avoid close personal contact, which facilitate fomite-mediated viral transmission including HRV, enteroviruses, AdV, and rotavirus [21]. Hence, the spread of HRV among children might be attributed to both the reduced effect of face masks and children’s uncontrolled behavior [22]. To be noted, RSV was reported to be the most common reason for LRTI-associated hospitalization in children less than 1 year of age, while HRV was reported to be the most common reason for LRTI-associated hospitalization in older children [20, 23], which might be due to the limited independent activity of children under 1 year of age.

Zhao et al.’s paper based on the respiratory samples of children in Shanghai during 2013–2015 shared some similar findings with ours, such as the age/gender preferences of HRV and the seasonality of HRV-C [24]. But HRVs were most frequently detected during winter in Zhao’s paper but summer in ours. In Zhao’s paper, the predominant genotypes included A78, A12, A89, A61, B70, C2, C6, C24 and C16, none of which were the main genotypes in our study. Moreover, we summarized the genotypes in papers focusing on various countries and concluded that the prevailing genotypes changed greatly with time and place [25–27]. But what these papers have in common was that HRV-As and HRV-Cs were the most frequently detected species and usually prevailed alternatively and seasonally. Considering of the substantial genetic diversity of HRVs, long-term and large-population-based studies are needed for a comprehensive understanding of HRV prevalence.

In consistence with our findings, several studies also reported that HRV-Cs are more commonly associated with early childhood asthma than the other two species [6, 28–30], which might be attributed to the different cellular receptors. HRV-As and HRV-Bs use intercellular adhesion molecule 1 (ICAM-1) and the low-density-lipoprotein receptor (LDLR) for viral binding [31–33], while Bochkov et al. found that HRV-Cs possibly use cadherin-related family member 3 (CDHR3) for viral binding [34]. Notably, CDHR3 is a susceptibility locus for wheezing illness and early childhood asthma [34]. Hence, anti-childhood wheezing and subsequent asthma control strategies should pay more attention to HRV-Cs.

It was reported that the patients co-infected with other respiratory viruses showed higher viral loads than those with HRV mono-infection [35], but it is not the case in our data. Also, the viral loads wasn’t correlated with the disease severity both in our study and other studies [36, 37], while the rates of underlying diseases increased progressively with disease severity, suggesting that host factors bear important responsibility for the disease severity. Notably, the viral load in nasopharyngeal swabs may not reflect the viral load in the lower respiratory tract, and the relationship between viral load in lower respiratory tract and severe LRTI deserves further exploration. Lee et al. reported that the detection rate and severity of HRV infections did not correspond, and HRV-As and HRV-Cs were more likely to develop severe LRTI than HRV-Bs [38], which is also the case in our data. A21 was more frequently detected in severe LRTIs than non-severe LRTIs and URTIs in our study, which is in line with the findings of a paper focusing on adults, although they didn’t find specific site mutations in the sequences of A21 obtained from severe cases [39]. Whether there are particular A21 mutations that facilitate viral replication and host adaptation, especially in the lower respiratory tract tropism, deserves to be further demonstrated.

There is a growing understanding on the pathogenesis of viral and bacterial coinfections. For instance, viral infection in the respiratory tract could induce airway damage, promote bacterial adherence, decrease mucociliary clearance and impair the immune system, all of which facilitate bacterial co-infection [40, 41]. Conversely, primary bacterial infection may predispose to viral infections by facilitating viral propagation and infection within the respiratory system [40]. In terms of the host’s factors, studies focusing on bacterial co-infections in COVID-19 patients found that advanced age and other comorbidities, such as chronic kidney disease, diabetes, and chronic heart disease, are associated with bacterial coinfections [42, 43]. But considering the small number of patients with bacterial co-infection (n = 22) in our study, we didn’t analyze the risk factors of bacterial co-infection in HRV-positive patients, which is a limitation of the study. There are also other limitations in this paper. For example, our data only collected the samples from children in 2020, which makes us fail to compare the epidemiological features of HRV genotypes before and after the outbreak of COVID-19. Moreover, genetic analysis is needed to figure out whether there are meaningful site mutations in the prevailing HRV genotypes, such as A21, A82 and A101. More efforts are needed for better understanding of the individual and viral factors that contribute to more severe illnesses, so as to reduce the overall burden of respiratory illness.

## Conclusions

Collectively, our findings described the details of the HRV prevalence among children in 2020, which is worthy of our reflection on the distinct transmitting pattern of HRVs. Moreover, our data suggested that the antiviral strategies to reduce HRV-related morbidity in high-risk children should focus on HRV-As and HRV-Cs. In a word, our findings add to the knowledge of the epidemiological features of HRV among children and underline the necessity to control HRV infection despite of the use of face masks.

## Data Availability

The datasets used and analysed during the current study are available from the corresponding author on reasonable request.

## Abbreviations

HRV: Human rhinoviruse
URTI: Upper respiratory tract infections
LRTI: Lower respiratory infection.
COVID-19: Coronavirus disease 2019
RSV: Respiratory syncytial virus
AdV: Adenovirus
IAV: Influenza A virus
IBV: Influenza B virus
PIV: Parainfluenza virus
MPV: Human metapneumovirus
ICAM-1: Intercellular adhesion molecule 1
LDLR: Low-density-lipoprotein receptor
CDHR3: Cadherin-related family member 3
